# Cytomegalovirus Diseases of the Gastrointestinal Tract

**DOI:** 10.3390/v14020352

**Published:** 2022-02-08

**Authors:** Pai-Jui Yeh, Ren-Chin Wu, Cheng-Tang Chiu, Ming-Wei Lai, Chien-Ming Chen, Yu-Bin Pan, Ming-Yao Su, Chia-Jung Kuo, Wey-Ran Lin, Puo-Hsien Le

**Affiliations:** 1 Department of Pediatric Gastroenterology, Chang Gung Memorial Hospital, Linkou Branch, Taoyuan 333, Taiwan; charlie01539@hotmail.com (P.-J.Y.); a22141@cgmh.org.tw (M.-W.L.); 2 Department of Pathology, Chang Gung Memorial Hospital, Linkou Branch, Taoyuan 333, Taiwan; renchin.wu@gmail.com; 3 Department of Gastroenterology and Hepatology, Chang Gung Memorial Hospital, Linkou Branch, Taoyuan 333, Taiwan; ctchiu@cgmh.org.tw (C.-T.C.); m7011@cgmh.org.tw (C.-J.K.); victor.wr.lin@gmail.com (W.-R.L.); 4 Taiwan Association of the Study of Small Intestinal Disease, Taoyuan 333, Taiwan; doctorsu@cgmh.org.tw; 5 Liver Research Center, Chang Gung Memorial Hospital, Linkou Branch, Taoyuan 333, Taiwan; 6 Department of Medical Imaging and Interventions, Chang Gung Memorial Hospital, Linkou Branch, Taoyuan 333, Taiwan; dr.cmchen@gmail.com; 7 Biostatistical Section, Clinical Trial Center, Chang Gung Memorial Hospital, Linkou Branch, Taoyuan 333, Taiwan; e8901145@gmail.com; 8 Department of Gastroenterology and Hepatology, New Taipei City Municipal Tucheng Hospital, New Taipei City 236, Taiwan

**Keywords:** cytomegalovirus, gastrointestinal tract, prognostic factor, antiviral therapy

## Abstract

Cytomegalovirus (CMV) infection of the gastrointestinal (GI) tract can be fatal. However, very few studies have provided comprehensive analyses and specified the differences in symptoms observed in different parts of the GI tract. This study aimed to comprehensively analyze clinical manifestations and management of GI CMV disease. This retrospective cohort study enrolled the patients who had CMV diseases of the GI tract proved by CMV immunohistochemistry stain from the pathology database in a 4000-bed tertiary medical center between January 2000 and May 2021. The patient characteristics, clinical manifestations, endoscopic features, treatments, outcomes, and prognostic factors were analyzed. A total of 356 patients were enrolled, including 46 infected in the esophagus, 76 in the stomach, 30 in the small intestine, and 204 in the colon. In total, 49.4% patients were immunocompromised. The overall in-hospital mortality rate was 20.8%: CMV enteritis had the highest rate (23.3%). Sixty percent of patients received antiviral treatment and 16% were administered both intravenous and oral anti-viral drugs (Combo therapy, minimal and mean treatment duration were 14 and 39.9 ± 25 days). Prognostic factors of in-hospital mortality included age, immune status, albumin level, platelet count, GI bleeding, time-to-diagnosis, and Combo therapy. In the survival analysis, immunocompetent patients receiving Combo therapy had the best survival curve, and immunocompromised patients receiving non-Combo therapy had the worst survival curve. Combo therapy ≥14 days resulted in a better outcome for both immunocompromised and immunocompetent patients. In conclusion, CMV GI diseases affect both immunocompromised and immunocompetent hosts, and a complete treatment course should be considered for patients with poor prognostic factors.

## 1. Introduction

Cytomegalovirus (CMV), a double-stranded DNA virus, is an important member of the *Herpesviridae* family [1]. CMV infection can manifest as asymptomatic, constitutional symptoms or tissue-invasive diseases [1,2]. The gastrointestinal (GI) tract is one of the most commonly involved systems and associated with 30% of tissue-invasive diseases among immunocompetent patients [3]. CMV GI disease is defined on the basis of upper and/or lower GI symptoms, macroscopic mucosal lesions, and CMV documented in tissue by histopathology, virus isolation, rapid culture, immunohistochemistry (IHC), or DNA hybridization techniques [4]. However, IHC staining has a higher sensitivity and specificity than routine HE staining [5,6]. Emerging research has shown that CMV diseases involved both immunocompromised and immunocompetent hosts [2,7,8,9]. The whole alimentary tract can be infected, but leading sites are the colon and esophagus [10]. CMV infection can worsen the outcomes of underlying GI diseases. For example, it increases the risk of hospitalization, colectomy, and even mortality in patients with inflammatory bowel disease (IBD) [1,11].

Studies regarding CMV disease of the whole GI tract were limited either by the small sample size or heterogeneous study population. Lin et al. reported the clinical and endoscopic features of alimentary tract CMV disease seen in 20 cases [12]. In adult patients with cancer, Ko et al. noted that male sex, low body mass index, lymphopenia, hematological malignancy, steroid use, and red blood cell transfusion within a month prior to the CMV disease diagnosis were independent risk factors for the development of CMV GI disease [13]. The two latest retrospective studies that enrolled 213 and 173 patients with positive H&E or IHC staining in Thailand found that antiviral treatment was the only protective factor that improved patient survival [7,9]. However, in our previous studies on CMV gastritis, enteritis, and colitis, antiviral therapy had no significant impact on in-hospital survival [8,14,15].

In this large retrospective study of GI CMV disease, we enrolled patients with GI CMV disease, confirmed by IHC staining, and aimed to investigate patient characteristics, clinical manifestations, endoscopic features, treatments, outcomes, prognostic factors of in-hospital mortality, and the differences in these factors observed at different sites.

## 2. Materials and Methods

### 2.1. Patient

We retrospectively enrolled patients with CMV diseases of the GI tract, confirmed by CMV immunohistochemistry staining, from the pathology database in a tertiary medical center between January 2000 and May 2021. The tissues were obtained via endoscopic biopsy or surgical incisions from sites between the esophagus and rectum. IHC staining was performed under either the clinician’s request or pathologist-initiated orders. CMV GI disease was diagnosed based on positive CMV IHC staining of the tissue, accompanied by clinical symptoms and endoscopic inflammatory changes. Immunohistochemical staining for CMV was performed on 3-um-thick paraffin sections with a mouse monoclonal antibody blend (1:200 dilution, clone 8B1.2/1G5.2/2D4.2, Zeta Corporation) on an automated stainer (BOND-MAX, Leica Biosystems, Wetzlar, Germany), then assessed with the BOND Polymer Refine Detection Kit (DS9800, Leica Biosystems). The results were interpreted as positive if there were any epithelial or mesenchymal cells exhibiting nuclear staining.

### 2.2. Data Collection

The medical records of eligible patients were reviewed for their age, sex, patient source (inpatient, outpatient), admission duration, date of diagnosis or recurrence, time-to-diagnosis, date of death or last follow-up, presence of critical conditions the week before diagnosis (shock, respiratory distress with ventilator usage), need for intensive care unit (ICU) care, underlying disease, medication history (corticosteroids, antibiotics), major clinical presentation, endoscopic findings (lesion characteristics, location, concomitant mucosal findings), histopathology results, laboratory results (total white blood cell (WBC), segment, lymphocyte, platelet (Plt), hemoglobin (Hb), creatinine (Cr), aspartate aminotransferase (AST), alanine aminotransferase (ALT), bilirubin, albumin, C-reactive protein (CRP) levels, CMV pp65 antigenemia, CMV viremia (Light-Mix^®^ Kit human cytomegalovirus (TIB Molbiol, Berlin, Germany, cut-off: Cp 35, 226 bp segment on glycoprotein B gene), COBAS^®^ AmpliPrep/COBAS^®^ TaqMan^®^ CMV Test (Roche Diagnostics, Branchburg, NJ, USA, cut-off: 150 copies/mL)), and CMV serology, treatments, complications, and outcomes (in-hospital and overall mortality). The laboratory and serology data were taken in the interval of 2 weeks before/after the date of diagnosis. The endoscopic features were categorized into three types: polypoid mass, ulcer, and inflammation (excluding concomitant masses or ulcers). GI bleeding was indicated by the presence of hematemesis, hematochezia, or melena. Recurrence is defined as a new tissue-proven CMV infection in a patient with previous CMV disease with a virus-free interval of at least 4 weeks [4]. Antiviral treatments were divided into three categories: IV (exclusively intravenous form), PO (exclusively oral form), and Combo (combined IV and PO forms).

### 2.3. Definition of Immune Status

Patients were defined as “immunocompromised” if they were recipients of solid organ or hematopoietic stem cell transplant or were documented to have primary immunodeficiency, human immunodeficiency virus infection, exposure to chemotherapeutic agents or radiotherapy within the last 6 months, and/or use immunosuppressants (including corticosteroids (oral or intravenous administration, ≥20 mg/day of prednisolone or any equivalent for >2 weeks)) [8,14,15].

### 2.4. Statistical Analysis

Numerical data are presented as mean ± standard deviation or median (interquartile range) values, while categorical data are expressed as absolute numbers and percentages. Independent t-tests and Mann–Whitney U tests were used to compare continuous variables, while χ^2^ and Fisher’s exact tests were used for categorical variables. Logistic regression models were used to identify the independent risk factors for in-hospital mortality. Statistical significance was set at *p* < 0.05. Results are presented as odds ratios (ORs), 95% confidence intervals (CIs), and *p*-values. Survival outcomes were evaluated using Kaplan–Meier survival curve analysis and log-rank test. For continuous parameters with statistical significance in multivariable logistic regression analysis, the optimal cut-off point with its sensitivity (Se) and specificity (Sp) were determined by receiver operating characteristic (ROC) curves using the Youden index method. All statistical calculations were performed using SPSS statistical software (Version 21.0; IBM Corp., Armonk, NY, USA).

### 2.5. Ethics

The study protocol conformed to the ethical guidelines of the 1975 Declaration of Helsinki and was approved by the Institutional Review Board (IRB) of the Chang Gung Medical Foundation (approval document No. 202101234B0. “Clinical presentations and outcome of cytomegalovirus, herpes simplex virus, Epstein-Barr virus, and Clostridioides difficile”) for the period 28 July 2021–27 July 2022. The Institutional Review Board does not require signed informed consent from individual patients to review medical records from the electronic medical record system in retrospective studies.

## 3. Results

### 3.1. Demographics and General Condition at Diagnosis

In Linkou Chang Gung Memorial Hospital, a 4000-bed tertiary referral center with 10,000 outpatients per day, 1448 GI tract specimens were examined for clinically suspected CMV diseases over two decades. A total of 356 eligible patients were enrolled in the study, with infection of the esophagus in 46, stomach in 76, small intestine in 30, and colon in 204 patients (Supplementary Figure S1). The average age was 60 years and did not differ widely among different locations. Male sex predominated (62.6%), especially in the esophagus group (78.3%). At the time of diagnosis, hospitalization was required in approximately 80% of patients; the general condition appeared worst in the small intestine group on the basis of highest percentage of critical illness (shock, intubation) and the need for intensive care (Table 1).

### 3.2. Underlying Diseases

**Figure 1:**
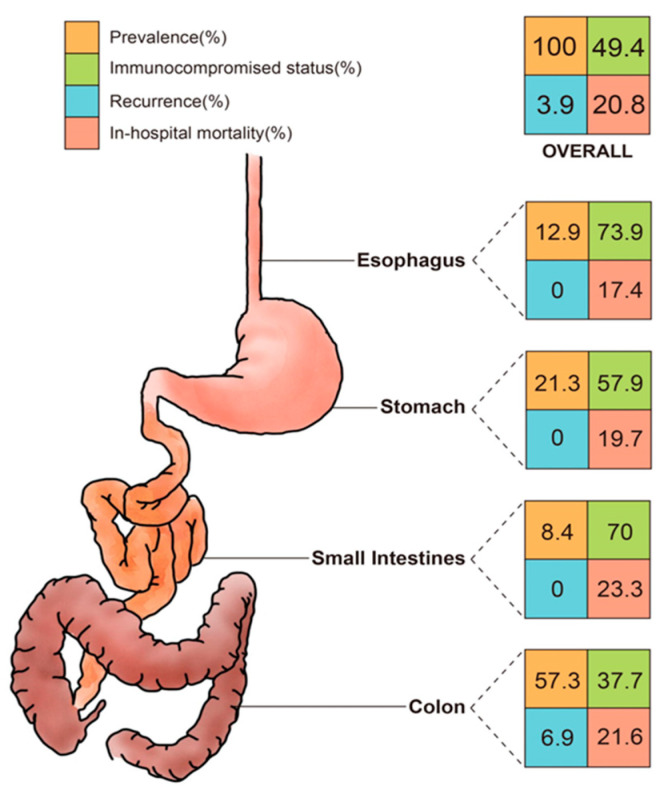
The percentages of prevalence, immunocompromised status, recurrence, and in-hospital mortality rates of CMV disease in different segments of the GI tract. CMV, cytomegalovirus; GI, gastrointestinal.

The leading underlying diseases were hypertension (45.5%) and diabetes mellitus (28.4%). IBD was found in approximately 10% patients of this cohort, mostly in the colon group. Malignancy was encountered in 27.5% of patients, with the highest percentage in the esophagus group (43.5%) and corresponded to the highest exposure to chemotherapy and radiotherapy. Overall, half (49.4%) of the cohort was immunocompromised; notably, the esophagus and small intestine group consisted of 73.9% and 70% of immunocompromised cases, respectively, which were substantially higher than the average (Figure 1).

### 3.3. Clinical Presentation and Diagnostic Work-Up

Initial presentations included GI bleeding (45.8%), fever (33.1%), and abdominal pain (31.5%). The small intestine group had a higher proportion of patients with these core symptoms. Some gastrointestinal symptoms, such as diarrhea, were specific to enterocolitis and were not compared in this study.

**Figure 2:**
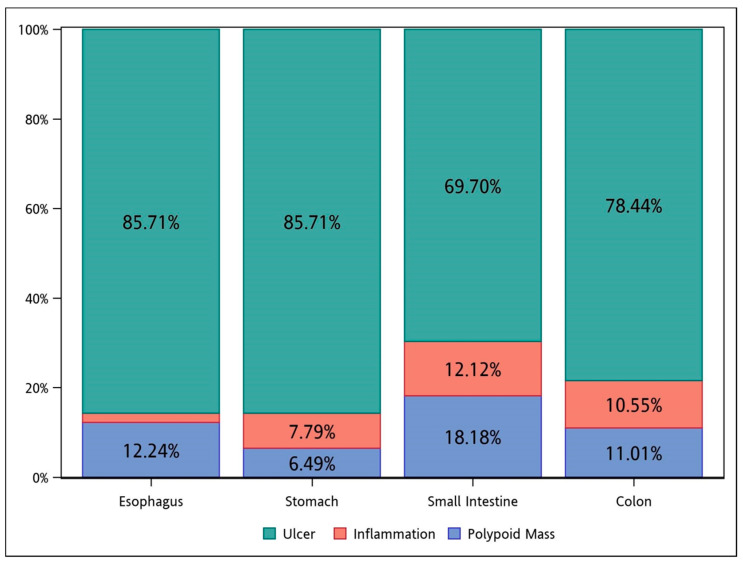
The endoscopic features of CMV diseases in different segments of the GI tract. CMV, cytomegalovirus; GI, gastrointestinal.

Endoscopically, ulcer was the major feature among all the groups (Figure 2). Inflammation, without concurrent ulcer or polypoid lesions, was observed in less than 15% of the cases in each group.

In laboratory examinations, patients predominately showed anemia, hypoalbuminemia, elevated CRP levels, and mildly elevated Cr levels. CMV status tests were not routinely performed for every patient, thus the results were relatively incomplete. The positive rates of CMV-IgG, CMV-IgM, antigenemia, and viremia were 97.2%, 18.3%, 54.8%, and 70.8%, respectively.

### 3.4. Treatment and Outcome

The mean duration of admission was 41.2 days. Eleven (3.1%) patients developed CMV-related GI tract perforation. Twenty-five (7%) patients required surgical treatment for CMV GI disease-associated indications, with the small intestine group accounting for almost three-fold of the average (20%).

Approximately 60% of patients received antiviral therapy, including oral (PO), intravenous (IV), or both forms sequentially (Combo). Ganciclovir was the solely utilized IV form agent, while valganciclovir and ganciclovir (four patients) were the two PO form agents documented. Only 16% of patients received Combo therapy; in this group, the minimal duration was 14 days, while the mean duration of IV form and the total duration were 14.9 ± 7.9 and 39.9 ± 25 days, respectively. Influenced by the individual’s clinical status (renal function, cytopenia, etc.), the dose and duration of antiviral therapy appeared heterogeneous and were difficult to compare and analyze.

The mean duration of follow-up was approximately 2.5 years. Fourteen cases (3.9%) of recurrence were observed in the colon group. The in-hospital mortality rate was 20.8% for the entire cohort, with the small intestine, colon, stomach, and esophagus groups ranked in descending order. The overall mortality rate was 40.4%.

### 3.5. Prognostic Factors of In-Hospital Mortality and Survival Analysis

In the univariable analysis, there were 20 risk factors and eight protective factors. Risk factors included age, shock, intubation, ICU, immunocompromised status, coronary artery disease, acute kidney injury, malignancy, chemotherapy, radiotherapy, steroid exposure, WBC, segment, CRP, fever, GI bleeding, ulcer (endoscopic feature), operation, exclusive IV therapy, and the time-to-diagnosis. Protective factors included outpatient treatment, ulcerative colitis, lymphocyte, hemoglobin, platelet, albumin, polypoid lesion (endoscopic feature), and Combo therapy. The multivariable analysis revealed seven independent prognostic factors, including age (OR 1.042, 95% CI 1.005–1.081; *p* = 0.026), immunocompromised status (OR 9.927, 95% CI 1.575–62.545; *p* = 0.015), albumin (OR 0.346, 95% CI 0.119–1.001; *p* = 0.050), platelet (OR 0.993, 95% CI 0.986–0.999; *p* = 0.027), GI bleeding (OR 6.067, 95% CI 1.611–22.84; *p* = 0.008), time-to-diagnosis (OR 1.023, 95% CI 1.003–1.044; *p* = 0.025), and Combo therapy (OR 0.031, 95% CI 0.002–0.589; *p* = 0.021) (Table 2). Using ROC analysis, the optimal cut-off values for continuous parameters were determined, including 54.5 years for age (Se = 82.4%, Sp = 37.6%), 152500 (/μL) for Plt (Se = 65.3%, Sp = 78.1%), 2.72 (g/dL) for albumin (Se = 83.6%, Sp = 54.8%), and 18.5 (days) for time-to-diagnosis (Se = 75.7%, Sp = 68.6%) (Supplementary Figure S2).

### 3.6. Impact of Different Locations, Treatment Courses, and Immune Status on In-Hospital Mortality

**Figure 3:**
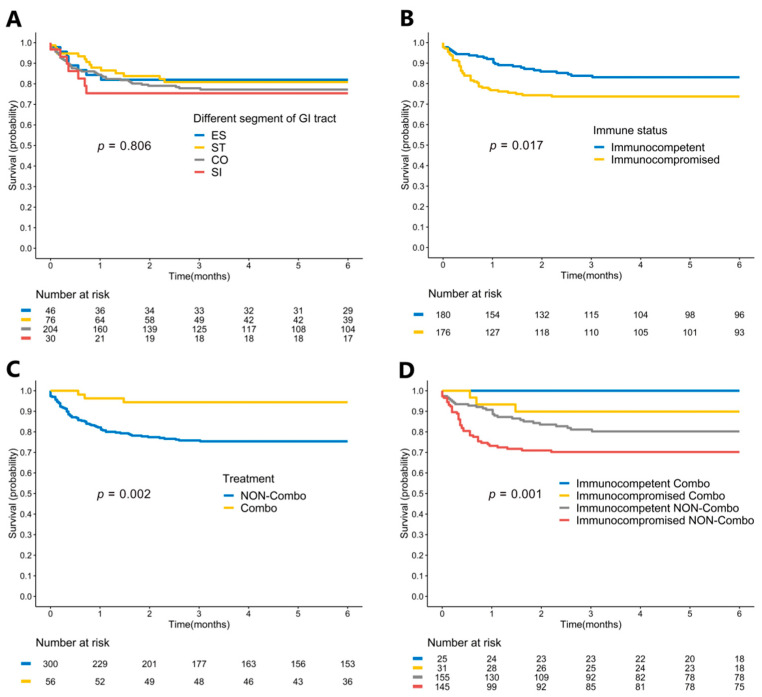
Kaplan–Meier survival curve analysis of patients with CMV GI diseases. (**A**) Patients with different infected locations had no statistical difference in in-hospital mortality rates (log-rank *p* = 0.806). (**B**) Patients with immunocompromised status had significantly worse survival outcomes than those with immunocompetent status (log-rank *p* = 0.017). (**C**) Patients receiving Combo therapy had significantly better survival outcomes than others (log-rank *p* = 0.002). (**D**) Patients with different immune status and treatment courses had significantly different survival outcomes (log-rank *p* = 0.001). CMV, cytomegalovirus; CO, colon; Combo, combination of intravenous and oral antiviral therapy; ES, esophagus; GI, gastrointestinal; SI, small intestine; ST, stomach.

In the Kaplan–Meier survival curve analysis, the patients at different infected locations were not significantly different with respect to the in-hospital survival curves (log-rank *p* = 0.806) (Figure 3A). Immunocompromised patients had significantly poorer survival curves than immunocompetent patients (log-rank *p* = 0.017) (Figure 3B). In contrast, patients who received Combo therapy had significantly better survival outcomes (log-rank *p* = 0.002) (Figure 3C). Overall, immunocompetent patients receiving Combo therapy had the best survival curve, and immunocompromised patients receiving non-Combo therapy had the worst survival curve (log-rank *p* = 0.001). (Figure 3D) Furthermore, the in-hospital mortality rates under different antiviral therapy courses were compared between immunocompetent and immunocompromised patients (Table 3). Anti-viral therapy (exclusive PO or IV) did not improve the in-hospital mortality, but Combo therapy (minimal duration ≥ 14 days) was related to a better outcome not only in immunocompromised patients but also in immunocompetent patients.

## 4. Discussion

This study comprised the largest number of cases of tissue-proven CMV GI diseases among similar studies in the literature, with all the enrolled cases prudently selected by positive IHC staining. Furthermore, this is the first study to provide detailed information on different segments of the alimentary tract, prognostic factors for in-hospital mortality, and the impact of different antiviral treatment courses.

CMV diseases, regardless of the end organs, are traditionally considered an infection primarily for immunocompromised patients. However, cohort studies of the GI tract in the past decades have composed a proportion of 25–50% of immunocompetent hosts [7,9,16]. Old age, critical illness, diabetes mellitus, chronic kidney disease, end-stage renal disease, and other comorbidities can lead to immune deficiency and increase the risk of CMV diseases [16,17,18]. However, these features traditionally do not define an immunocompromised status. Clinical physicians should keep the diagnosis in mind when this high-risk group of patients present with relative symptoms.

The diagnosis of CMV GI diseases is challenging because of the diverse presentations, endoscopic findings, biopsy locations, and laboratory methods. Symptoms and laboratory parameters are not distinguishable from other etiologies of infectious diseases. Variable ulcers are the most common endoscopic features of CMV infection; however, diagnosis based on endoscopic findings is difficult [17,19,20]. Although serology tests provide a hint of CMV diseases, their results correlate inadequately to the presence and severity of CMV tissue invasion; hence, histopathology remains the gold standard to confirm the tissue invasion by CMV in an inflammatory background [4,21]. However, the percentage of CMV viremia was relatively low in this study. In our hospital, CMV IHC staining was widely used in clinically or pathologically suspicious cases. In this way, we might identify more mild GI CMV disease without viremia. Compared to H&E staining, IHC staining provides higher sensitivity and specificity [1,22]. We only enrolled patients with CMV IHC staining confirmation; thus, the criterion is stricter and more rigorous than in previous studies. Although quantitative polymerase chain reaction has diagnostic accuracy similar to that of IHC staining in some studies, it has not been widely applied in our institution [23,24].

In this study, the prevalence of CMV enteritis (8.4%) was the lowest, but it was associated with the worst in-hospital survival rate. Difficult tissue sampling and a higher cost of enteroscopy may lead to missed and delayed diagnosis. In addition, the highest percentage of patients having immunocompromised status, critical illness, perforation, and surgery, also played important roles. Therefore, we should be aware of CMV enteritis in this group of high-risk patients with unexplained fever, abdominal pain, or GI bleeding.

In this cohort, seven negative prognostic factors of in-hospital mortality were identified and could be classified into three aspects: host status (old age, immunocompromised status, hypoalbuminemia), manifestation (GI bleeding, thrombocytopenia), and intervention (longer time-to-diagnosis duration, non-Combo therapy). In host status, old age, immunocompromised status, and malnutrition (hypoalbuminemia) resulted in impaired immune function and then poor survival. In two prior studies, old age and malnutrition were noted as negative predictive factors for mortality as well [7,9]. Since hypoalbuminemia indicates malnourishment, poor immunity, and worse tissue healing, it is responsible for increased mortality rates in several diseases, especially in patients in the ICU setting [25,26,27]. Thus, nutritional assessment and support are crucial for improving the survival rate of patients with GI CMV disease. With regard to immunocompromised status, Wetwittayakhlang et al. reported no significant difference in in-hospital survival, while Chaemsupaphan et al. noted that the six-month mortality was higher in immunocompetent patients. These discrepant results might be associated with differences in patient selection (IHC staining, definition of immunocompromised status), study endpoints (disease-specific mortality or overall mortality), and treatment strategies [7,9]. Second, thrombocytopenia and GI bleeding were negative predictors of in-hospital mortality. The optimal cut-off value for platelet count (152500 cells/μL) in this study was close to the lower limit of the reference range, paralleling the ordinary interpretation of thrombocytopenia [28]. Thrombocytopenia could be caused by viral effects (direct injury to multipotent stem cells, hemophagocytosis, and immunological mechanisms), sepsis with disseminated intravascular coagulation, and hematologic toxicity of antiviral agents [29]. If patients with GI CMV disease present with thrombocytopenia and GI bleeding, it may imply more severe diseases. Finally, the type of intervention did matter. In view of the nonspecific clinical, laboratory, or endoscopic presentations, lower awareness of CMV diseases in immunocompetent patients, and lower sensitivity of H&E staining for CMV inclusion bodies, definite diagnosis of CMV disease might be delayed and may postpone further management. In our study, the optimal cut-off of time-to-diagnosis in the survival analysis was 18.5 days; however, there was scarce relevant data in the literature to compare it with.

Once the diagnosis is confirmed, the dosage or prescription of immunosuppressive drugs and steroids could be reduced or stopped, respectively. Although the efficacy and benefit of antiviral agents for general CMV diseases in patients undergoing organ transplantation and with HIV infection have been addressed in reviews and guidelines, they are still controversial in other conditions [30,31]. Most recommendations were made for immunocompromised populations, while the evidence for immunocompetent populations was limited [2,3,10,21,32]. Two studies mentioned that anti-viral therapies improved the in-hospital survival in both immunocompetent and compromised patients with 14 and 21 day therapeutic durations [7,9]. In other review articles, they suggested antiviral treatment for at least 2–3 weeks [2,22]. In this study, we found that Combo therapy (IV + PO) (minimal duration: 2 weeks, average duration: 5 weeks) resulted in better in-hospital survival rates in both immunocompetent and immunocompromised groups. Patients who received both IV and PO anti-viral agents tended to have a more complete therapeutic course than others. Nevertheless, the patients who received only IV form of anti-viral agents had a higher mortality rate in both groups; they received only IV drugs without extended oral antiviral agents, which might be due to their critical condition. Additionally, side effects of antiviral agents, including acute kidney injury and pancytopenia, might have given rise to poorer outcomes. This was the first study to compare the survival of patients with different immune statuses and treatment courses.

The limitations of this study include its retrospective design, the evolvement of clinical practice (awareness of diagnosis in immunocompetent patients and inflammatory bowel disease), diagnostic tools (accessibility of CMV IHC staining, single-balloon enteroscopy, and double-balloon enteroscopy), incomplete CMV status tests, and heterogeneous data of antiviral therapy. In Combo therapy, the patients had a continuous treatment course from IV to PO antiviral agents, and it was easier to record the therapeutic duration. On the other hand, intermittent medication due to intolerance, impaired renal function, and myelosuppression were frequently noted in exclusive IV or PO treatment groups, and we could not analyze the exact therapeutic duration in these patients.

## 5. Conclusions

In CMV GI diseases, up to 50% of patients were immunocompetent, leading to 21% in-hospital and 40% overall mortality rates. Although the colon was the most commonly involved location, CMV enteritis had the worst outcome. Among the seven independent prognostic factors, immune status and antiviral treatment significantly influenced survival. With good awareness and a complete treatment course, we might improve the outcomes of GI CMV diseases.

## Figures and Tables

**Table 1:** Characteristics of different segments of CMV GI diseases.

Characteristics	ES (*n* = 46)	ST (*n* = 76)	SI (*n* = 30)	CO (*n* = 204)	All (*n* = 356)
Sex (M/F)	36 (78.3%)	45 (59.2%)	20 (66.7%)	122 (59.8%)	223 (62.6%)
Age, year (mean ± SD)	59.7 ± 18.1	59.1 ± 17.8	50 ± 21	61.9 ± 18.3	60 ± 18.6
General condition					
OPD/IPD	11 (23.9%)	30 (39.5%)	3 (10%)	38 (18.6%)	82 (23%)
Shock	8 (17.4%)	9 (11.8%)	10 (33.3%)	47 (23%)	74 (20.8%)
Intubation	6 (13%)	7 (9.2%)	8 (26.7%)	46 (22.5%)	67 (18.8%)
ICU	7 (15.2%)	13 (17.1%)	10 (33.3%)	58 (28.4%)	88 (24.7%)
Underlying disease					
Immunocompromised	34 (73.9%)	44 (57.9%)	21 (70%)	77 (37.7%)	176 (49.4%)
DM	9 (19.6%)	23 (30.3%)	3 (10%)	66 (32.4%)	101 (28.4%)
HTN	20 (43.5%)	38 (50%)	10 (33.3%)	94 (46.1%)	162 (45.5%)
Old CVA	4 (8.7%)	4 (5.3%)	2 (6.7%)	31 (15.2%)	41 (11.5%)
COPD	4 (8.7%)	2 (2.6%)	2 (6.7%)	10 (4.9%)	18 (5.1%)
CAD	5 (10.9%)	5 (6.6%)	3 (10%)	32 (15.7%)	45 (12.6%)
LC	2 (4.3%)	6 (7.9%)	0 (0%)	8 (3.9%)	16 (4.5%)
ESRD	4 (8.7%)	8 (10.5%)	6 (20%)	25 (12.3%)	43 (12.1%)
AKI	6 (13%)	8 (10.5%)	7 (23.3%)	48 (23.5%)	69 (19.4%)
CD	0 (0%)	0 (0%)	3 (10%)	8 (3.9%)	11 (3.1%)
UC	1 (2.2%)	1 (1.3%)	1 (3.3%)	31 (15.2%)	34 (9.6%)
HIV	8 (17.4%)	5 (6.6%)	2 (6.7%)	18 (8.8%)	33 (9.3%)
Malignancy	20 (43.5%)	29 (38.2%)	11 (36.7%)	38 (18.6%)	98 (27.5%)
Transplant	3 (6.5%)	5 (6.6%)	5 (16.7%)	7 (3.4%)	20 (5.6%)
Chemotherapy	15 (32.6%)	23 (30.3%)	5 (16.7%)	18 (8.8%)	61 (17.1%)
Radiotherapy	16 (34.8%)	14 (18.4%)	5 (16.7%)	11 (5.4%)	46 (12.9%)
Steroid	23 (50%)	36 (47.4%)	13 (43.3%)	65 (31.9%)	137 (38.5%)
Immunosuppressant	6 (13%)	11 (14.7%)	10 (33.3%)	17 (8.3%)	44 (12.4%)
Laboratory data (mean ± SD)					
WBC	6471.4 ± 4592.8	7743.9 ± 4697.1	7742.9 ± 3840.2	8176.2 ± 4179.1	7837 ± 4329.9
Segment	76.2 ± 13.9	70.7 ± 15.8	71.3 ± 18.5	73.9 ± 13.8	73.3 ± 14.7
Lymphocyte	12.6 ± 10.3	18 ± 14.5	16.1 ± 14.1	16.7 ± 12	16.4 ± 12.6
Hemoglobin	10.3 ± 1.7	10.2 ± 2	9.2 ± 2.8	10.4 ± 2.6	10.2 ± 2.5
Platelet	186.2 ± 91.8	197.3 ± 111.9	198.4 ± 110.6	235.8 ± 128.7	218.5 ± 121.1
Bilirubin	0.7 ± 0.5	1.8 ± 7.6	0.9 ± 1	1.3 ± 2.8	1.3 ± 4.1
Creatinine	1.4 ± 1.6	1.8 ± 3.7	2.1 ± 2.1	1.8 ± 2.2	1.8 ± 2.5
Albumin	2.7 ± 0.7	3.1 ± 0.7	2.8 ± 0.6	3 ± 3.8	2.9 ± 2.9
CRP	57.7 ± 61.7	61.1 ± 74.6	76.9 ± 76.5	62.6 ± 71.3	63.3 ± 71.3
CMV status					
CMV IgM	3 (21.4%)	7 (20%)	4 (26.7%)	14 (15.7%)	28 (18.3%)
CMV IgG	13 (92.9%)	29 (96.7%)	15 (100%)	83 (97.6%)	140 (97.2%)
CMV antigenemia	8 (66.7%)	13 (48.1%)	4 (36.4%)	43 (58.1%)	68 (54.8%)
CMV viremia	4 (57.1%)	6 (33.3%)	8 (72.7%)	57 (81.4%)	75 (70.8%)
Clinical presentation					
Fever	17 (37%)	20 (26.3%)	12 (40%)	69 (33.8%)	118 (33.1%)
Abdominal pain	20 (43.5%)	30 (39.5%)	15 (50%)	47 (23%)	112 (31.5%)
GI bleeding	14 (30.4%)	26 (34.2%)	21 (70%)	102 (50%)	163 (45.8%)
Endoscopic feature					
Polypoid mass	6 (13%)	5 (6.6%)	6 (20%)	24 (11.8%)	41 (11.5%)
Inflammation	1 (2.2%)	6 (7.9%)	4 (13.3%)	23 (11.3%)	34 (9.6%)
Ulcer	42 (91.3%)	66 (86.8%)	23 (76.7%)	171 (83.8%)	302 (84.8%)
Treatment					
Operation	0 (0%)	2 (2.6%)	6 (20%)	17 (8.3%)	25 (7%)
IV ± PO	27 (58.7%)	40 (52.6%)	18 (60%)	127 (62.3%)	212 (59.6%)
IV + PO (Combo)	6 (13%)	10 (13.2%)	6 (20%)	34 (16.7%)	56 (15.7%)
IV (exclusive)	8 (17.4%)	12 (15.8%)	10 (33.3%)	55 (27%)	85 (23.9%)
PO (exclusive)	13 (28.3%)	17 (22.4%)	2 (6.7%)	37 (18.1%)	69 (19.4%)
Course/Outcome (mean ± SD)					
Time-to-diagnosis	15.8 ± 13.1	17.8 ± 20.1	19.7 ± 16.3	21.5 ± 21.1	19.8 ± 19.7
Admission duration	33.4 ± 27.2	41.9 ± 34.5	32 ± 18	44.2 ± 34.5	41.2 ± 32.6
Follow up duration	922.1 ± 1504.8	1532.6 ± 5139.1	637.6 ± 1049.7	768.8 ± 1199.3	939.8 ± 2615.1
Perforation	0 (0%)	1 (1.3%)	2 (6.7%)	8 (3.9%)	11 (3.1%)
Recurrence	0 (0%)	0 (0%)	0 (0%)	14 (6.9%)	14 (3.9%)
In-hospital mortality	8 (17.4%)	15 (19.7%)	7 (23.3%)	44 (21.6%)	74 (20.8%)
Overall mortality	23 (50%)	31 (40.8%)	13 (43.3%)	77 (37.7%)	144 (40.4%)

Abbreviations: AKI, acute kidney injury; CAD, coronary artery disease; CD, Crohn’s disease; CMV, cytomegalovirus; CO, colon; COPD, chronic obstructive pulmonary disease; CRP, C reactive protein; CVA, cardiovascular accident; DM, diabetes mellitus; ES, esophagus; ESRD, end stage renal disease; F, female; GI, gastrointestinal; HIV, human immunodeficiency virus; HTN, hypertension; ICU, intensive care unit; IPD, inpatient; IV, intravenous; LC, liver cirrhosis; M, male; OPD, outpatient; PO, oral; SD, standard deviation; SI, small intestine; ST, stomach; UC, ulcerative colitis; WBC, white blood cell.

**Table 2:** Prognostic factors of in-hospital mortality for CMV GI diseases.

Characteristics	Univariable Analysis			Multivariable Analysis		
	OR	95% CI	*p*-Value	OR	95% CI	*p*-Value
Sex (M/F)	0.844	0.5–1.425	0.525			
Age, year	1.023	1.007–1.039	0.004 *	1.042	1.005–1.081	0.026 *
General condition						
OPD source	0.034	0.005–0.249	0.001 *	<0.001	-	0.999
Shock	6.622	3.737–11.732	<0.001 *	1.754	0.571–5.39	0.326
Intubation	4.926	2.76–8.792	<0.001 *	1.247	0.289–5.388	0.768
ICU	7.305	4.168–12.803	<0.001 *	2.078	0.536–8.053	0.290
Underlying disease						
Immunocompromised	1.921	1.136–3.247	0.015 *	9.927	1.575–62.545	0.015 *
DM	0.843	0.471–1.507	0.564			
HTN	1.440	0.862–2.407	0.164			
Old CVA	1.467	0.697–3.087	0.313			
COPD	0.752	0.212–2.67	0.66			
CAD	2.407	1.227–4.721	0.011 *	2.040	0.511–8.136	0.313
LC	0.532	0.118–2.393	0.411			
ESRD	1.010	0.461–2.212	0.98			
AKI	3.283	1.846–5.838	<0.001 *	1.799	0.564–5.737	0.321
CD	<0.001	-	-			
UC	0.217	0.051–0.927	0.039	2.677	0.178–40.367	0.477
HIV	0.355	0.105–1.197	0.095			
Malignancy	2.313	1.352–3.958	0.002 *	2.692	0.335–21.653	0.352
Transplant	0.951	0.308–2.933	0.93			
Chemotherapy	1.974	1.066–3.655	0.03 *	0.601	0.088–4.12	0.604
Radiotherapy	2.602	1.339–5.055	0.005 *	2.157	0.309–15.038	0.438
Steroid	2.407	1.431–4.05	0.001 *	0.866	0.232–3.238	0.831
Immunosuppressant	0.689	0.294–1.615	0.392			
Laboratory data						
WBC	1.000	1.000–1.000	0.006 *	1.000	1.000–1.000	0.075
Segment	1.067	1.041–1.094	<0.001 *	0.957	0.858–1.068	0.435
Lymphocyte	0.920	0.889–0.951	<0.001 *	0.951	0.843–1.072	0.411
Hemoglobin	0.783	0.683–0.898	<0.001 *	0.938	0.714–1.232	0.644
Platelet	0.992	0.989–0.995	<0.001 *	0.993	0.986–0.999	0.027 *
Bilirubin	1.058	0.975–1.148	0.173			
Creatinine	1.055	0.961–1.158	0.264			
Albumin	0.233	0.134–0.405	<0.001 *	0.346	0.119–1.001	0.050 *
CRP	1.010	1.006–1.014	<0.001 *	1.003	0.996–1.009	0.433
Clinical presentation						
Fever	2.152	1.275–3.631	0.004 *	1.848	0.603–5.664	0.283
Abdominal pain	0.903	0.517–1.577	0.719			
GI bleeding	4.663	2.626–8.28	<0.001 *	6.067	1.611–22.84	0.008 *
Endoscopic feature						
Polypoid mass	0.271	0.081–0.905	0.034 *	0.325	0.025–4.202	0.390
Inflammation	0.632	0.236–1.694	0.362			
Ulcer	2.902	1.113–7.567	0.029 *	1.631	0.268–9.933	0.596
Treatment						
Operation	2.781	1.194–6.477	0.018 *	0.951	0.165–5.463	0.955
IV ± PO	1.328	0.78–2.263	0.296			
IV + PO (Combo)	0.183	0.055–0.602	0.005 *	0.031	0.002–0.589	0.021 *
IV (exclusive)	4.164	2.404–7.212	<0.001 *	1.009	0.324–3.142	0.988
PO (exclusive)	0.591	0.286–1.22	0.155			
Course/Outcome						
Time-to-diagnosis	1.032	1.016–1.048	<0.001 *	1.023	1.003–1.044	0.025 *
Perforation	0.843	0.178–3.986	0.829			

Abbreviations: AKI, acute kidney injury; CAD, coronary artery disease; CD, Crohn’s disease; CI, confidence interval; CMV, cytomegalovirus; COPD, chronic obstructive pulmonary disease; CRP, C reactive protein; CVA, cardiovascular accident; DM, diabetes mellitus; ESRD, end stage renal disease; F, female; GI, gastrointestinal; HIV, human immunodeficiency virus; HTN, hypertension; ICU, intensive care unit; IV, intravenous; LC, liver cirrhosis; M, male; OPD, outpatient; OR, odds ratio; PO, oral; UC, ulcerative colitis; WBC, white blood cell; * *p* ≦ 0.05, calculated by logistic regression analysis.

**Table 3:** The impact of different antiviral treatment courses and immune status on in-hospital mortality in CMV GI diseases.

	Immunocompetent (*n* = 180)			Immunocompromised (*n* = 176)		
	Survival	Death	*p*-Value	Survival	Death	*p*-Value
Any treatment (IV or PO)	81 (53.3%)	18 (64.3%)	0.282	83 (63.8%)	30 (65.2%)	0.868
No treatment (IV or PO)	71 (46.7%)	10 (35.7%)		47 (36.2%)	16 (34.8%)	
IV + PO (Combo) (+)	25 (16.4%)	0 (0%)	0.016 *	28 (21.5%)	3 (6.5%)	0.022 *
IV + PO (Combo) (−)	127 (83.6%)	28 (100%)		102 (78.5%)	43 (93.5%)	
IV (exclusive) (+)	24 (15.8%)	14 (50%)	<0.001 *	26 (20%)	21 (45.7%)	0.001 *
IV (exclusive) (−)	128 (84.2%)	14 (50%)		104 (80%)	25 (54.3%)	
PO (exclusive) (+)	31 (20.4%)	4 (14.3%)	0.453	28 (21.5%)	6 (13%)	0.21
PO (exclusive) (−)	121 (79.6%)	24 (85.7%)		102 (78.5%)	40 (87%)	

Abbreviations: CMV, cytomegalovirus; GI, gastrointestinal; IV, intravenous; PO, oral; * *p* ≦ 0.05, calculated by Chi-square or Fisher’s exact test on categorical data.

## Data Availability

The datasets during and/or analyzed during the current study are available from the corresponding author on reasonable request.

## Supplementary Materials

The following are available online at www.mdpi.com/article/10.3390/v14020352/s1. Figure S1: Trends of annual case numbers of cytomegalovirus gastrointestinal diseases. Figure S2: Receiver operating characteristic analysis of cut-off values for continuous parameters, (A) age; (B) platelet, (C) albumin, (D) time-to-diagnosis.
