# Supplementary material for: Cytomegalovirus Diseases of the Gastrointestinal Tract
Source: Viruses. 2022 Feb 8;14(2):352. doi: 10.3390/v14020352 (PMC8879032; doi:10.3390/v14020352)
Supplement: Supplementary file 1 [file viruses-14-00352-s001.zip › viruses-1543768-supplementary.pdf]

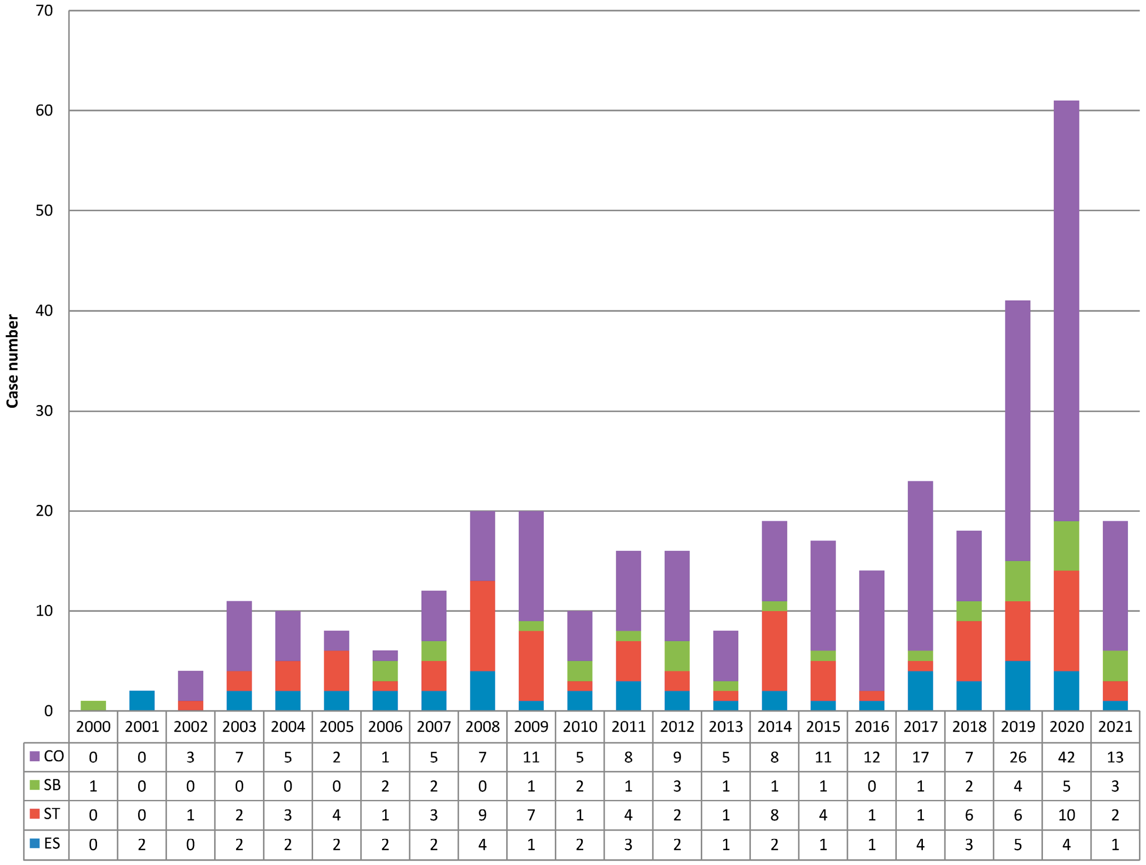

	2000	2001	2002	2003	2004	2005	2006	2007	2008	2009	2010	2011	2012	2013	2014	2015	2016	2017	2018	2019	2020	2021
CO	0	0	3	7	5	2	1	5	7	11	5	8	9	5	8	11	12	17	7	26	42	13
SB	1	0	0	0	0	0	2	2	0	1	2	1	3	1	1	1	0	1	2	4	5	3
ST	0	0	1	2	3	4	1	3	9	7	1	4	2	1	8	4	1	1	6	6	10	2
ES	0	2	0	2	2	2	2	2	4	1	2	3	2	1	2	1	1	4	3	5	4	1

Figure S1. Trends of annual case numbers of cytomegalovirus gastrointestinal diseases.

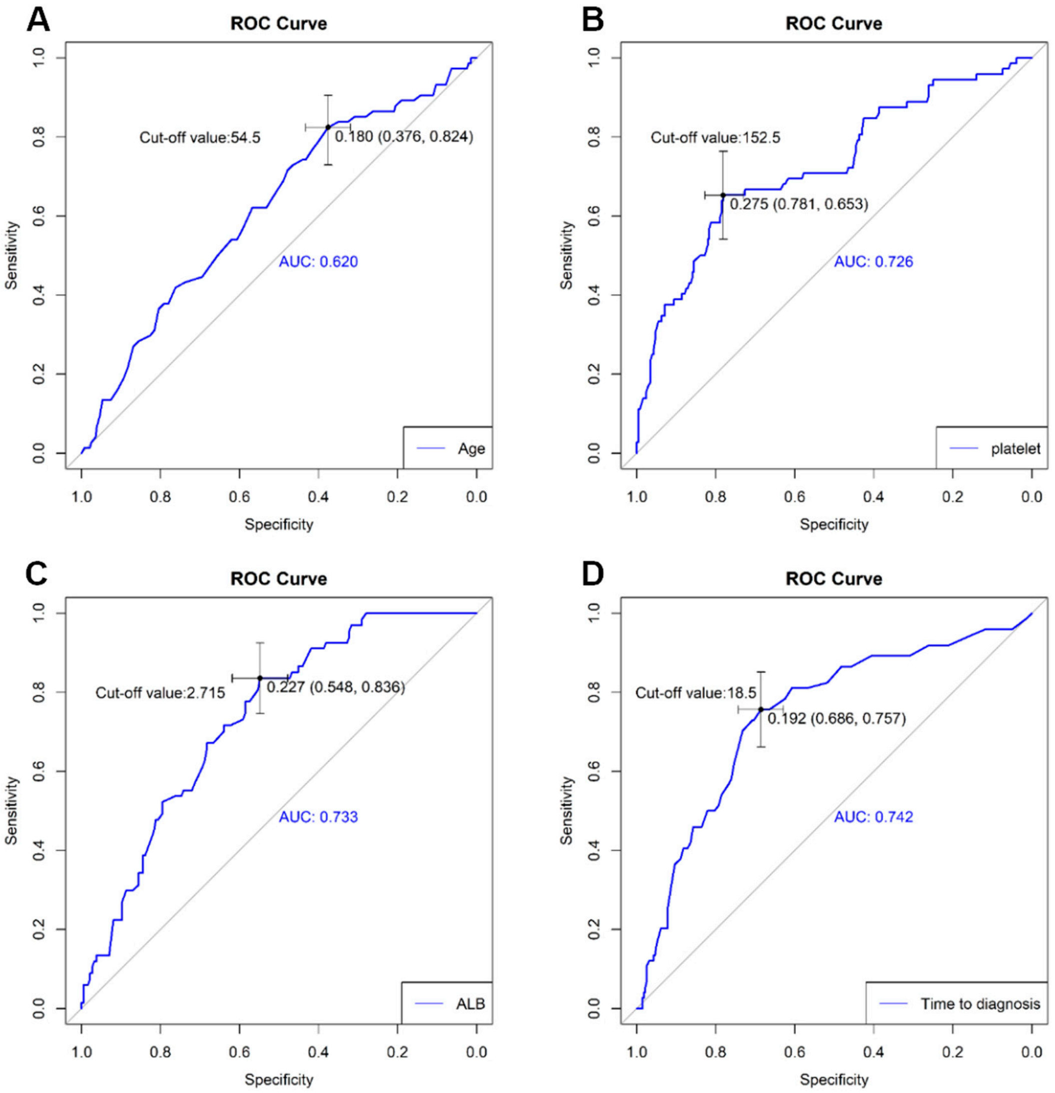

Figure S2. Receiver operating characteristic analysis of cut-off values for continuous parameters, (**A**) age; (**B**) platelet, (**C**) albumin, (**D**) time-to-diagnosis.
